# Sequential Approach for Water Purification Using Seashell-Derived Calcium Oxide through Disinfection and Flocculation with Polyphosphate for Chemical Pollutant Removal

**DOI:** 10.1021/acsomega.3c07627

**Published:** 2024-03-06

**Authors:** Yuuki Hata, Sumiyo Hiruma, Hiromi Miyazaki, Shingo Nakamura

**Affiliations:** † Department of Chemical Science and Engineering, School of Materials and Chemical Technology, Tokyo Institute of Technology, 2-12-1-H121 Ookayama, Meguro-ku 152-8550, Tokyo, Japan; ‡ Division of Biomedical Engineering, National Defense Medical College Research Institute, 3-2 Namiki, Tokorozawa-shi 359-8513, Saitama, Japan

## Abstract

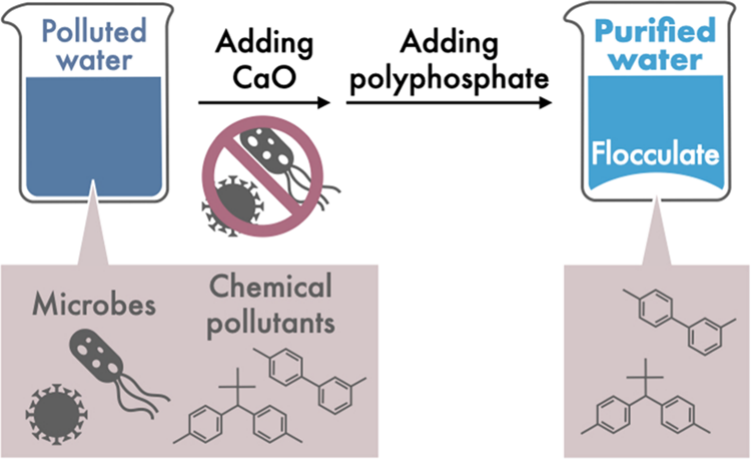

Safe water supply is usually inadequate in areas without water treatment plants and even in a city under emergency conditions due to a disaster, even though safe water is essential for drinking and other various purposes. The purification of surface water from a river, lake, or pond requires disinfection and removal of chemical pollutants. In this study, we report a water purification strategy using seashell-derived calcium oxide (CaO) via disinfection and subsequent flocculation with polyphosphate for chemical pollutant removal. Seashell-derived CaO at a concentration (2 g L^–1^) higher than its saturation concentration caused the >99.999% inactivation of bacteria, mainly due to the alkalinity of calcium hydroxide (Ca(OH)_2_) produced by hydration. After the disinfection, the addition of sodium polyphosphate at 2 g L^–1^ allowed for the flocculation of CaO/Ca(OH)_2_ particles with adsorbing chemical pollutants, such as Congo red, dichlorodiphenyltrichloroethane, di(2-ethylhexyl)phthalate, and polychlorinated biphenyls, for removing these pollutants; purified water was obtained through filtration. Although this purified water was initially highly alkaline (pH ∼ 12.5), its pH decreased into a weak alkaline region (pH ∼ 9) during exposure to ambient air by absorbing carbon dioxide from the air with the precipitating calcium carbonate. The advantages of this water purification strategy include the fact that the saturation of CaO/Ca(OH)_2_ potentially serves as a visual indicator of disinfection, that the flocculation by polyphosphate removes excessive CaO/Ca(OH)_2_ as well as chemical pollutants, and that the high pH and Ca^2+^ concentrations in the resulting purified water are readily decreased. Our findings suggest the usability of seashell-derived material–polymer assemblies for water purification, especially under emergency conditions due to disasters.

## 1 Introduction

Safe and purified water is essential for drinking and other various purposes. Its supply is normally sufficient in developed countries but would be inadequate in areas without water treatment plants and even in a city under emergency conditions due to a disaster. The only available water can be surface water from the river, lake, or pond that contains chemical pollutants and harmful microorganisms. Chemical pollutants can be removed by flocculation, the use of adsorbents (e.g., activated carbon), and filtration.^1−6^ Moreover, water disinfection is achieved by heating, filtration, and the use of disinfectants, such as hypochlorite and iodine.^1,7−9^ The use of disinfectants has some advantages, including the ease of application to large or small quantities of water and no requirement for electricity or fuel sources. Nevertheless, appropriate control over the concentration of the disinfectants is required. In fact, necessary amounts of halogen-based disinfectants (e.g., hypochlorite) increase with the presence of organic contaminants, as these disinfectants are consumed in a reaction with organic compounds produced from the decomposition of organisms and their wastes.^7,8^ Furthermore, a too high concentration of halogen-based disinfectants causes objectionable taste and smell and might generate carcinogenic compounds (e.g., trihalomethane).^7−9^ Overall, each water treatment method has different advantages and disadvantages and is used for different purposes and situations. Thus, the development of a novel water treatment method will increase our accessibility to safe water.

Seashell-derived calcium oxide (CaO) has recently attracted the attention of researchers as a sustainable raw material for safe disinfectants.^10,11^ Seashells are industrial wastes accumulating on the shores of harvesting districts and cause offensive odor, soil pollution, and other environmental problems; their utilization is highly desired from the viewpoint of sustainability.^10^ In this context, seashell-derived CaO, which is prepared via heat treatments of seashells composed mainly of calcium carbonate (CaCO_3_), has been used as a food additive for pH control, nutrient supplement, dough conditioning, and other purposes.^12,13^ Moreover, studies have investigated its application as a disinfectant with excellent microbicidal and virucidal activities.^10,11^ The microbicidal and virucidal activities originate mainly from the alkalinity of calcium hydroxide (Ca(OH)_2_) produced from CaO via hydration, while reactive oxygen species generated by CaO particles may also contribute.^10,14,15^ Seashell-derived CaO has been shown to exhibit a broad spectrum of microbicidal and virucidal activities against various pathogenic bacteria,^15^ viruses,^16^ bacterial spores,^17,18^ fungi,^19^ and biofilms,^20−22^ and its aqueous solutions and suspensions have been investigated for the disinfection of food, such as vegetables,^23,24^ meats,^25,26^ and fish.^27^ Moreover, we have recently explored the medical use of these water-based disinfectants from seashell-derived CaO.^11,28−32^ Given that it is a common food additive, seashell-derived CaO is a promising disinfectant for producing safe and pure water that is usable for drinking and even medical purposes; however, the potential of seashell-derived CaO for use in water treatments has rarely been investigated so far.

**Figure 1:**
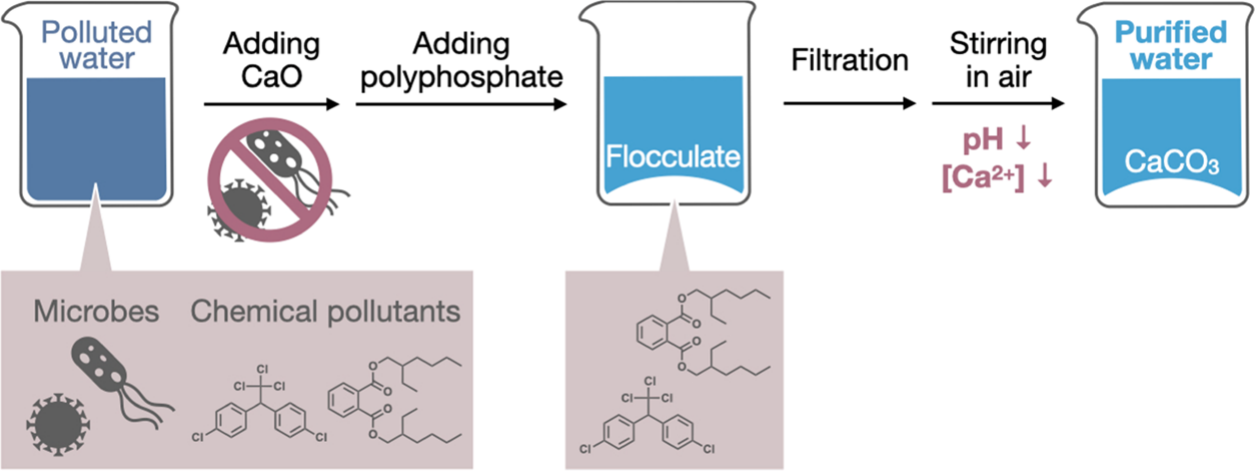
Schematic illustration of the water purification process using seashell-derived CaO via disinfection and subsequent flocculation with polyphosphate involving chemical pollutants.

Herein, we demonstrate the use of seashell-derived CaO not only for disinfection but also for the removal of chemical pollutants from contaminated water using polyphosphate (Figure 1). This study aimed to investigate the effectiveness of seashell-derived CaO for water purification. A model-contaminated water was prepared by adding a bacterium, *Escherichia coli* (*E. coli*), and chemical pollutants, such as dichlorodiphenyltrichloroethane (DDT), di(2-ethylhexyl)phthalate (DEHP), and polychlorinated biphenyls (PCBs), to ultrapure water. Seashell-derived CaO was added to this contaminated water at a concentration higher than its saturation concentration, which led to the >99.999% inactivation of *E. coli*. After the disinfection by seashell-derived CaO, anionic polyphosphate, which is widely used as a food additive,^33^ was added for the flocculation of the CaO/Ca(OH)_2_ particles with a positive surface charge, as previously reported.^34^ Notably, this flocculation occurred, involving the chemical pollutants, namely, DDT, DEHP, and PCBs, in water; the removal of aggregates by filtration resulted in the production of purified water. Moreover, although this purified water was initially highly alkaline (pH ∼ 12.5), its pH decreased into the weak alkaline region (pH ∼ 9) during exposure to ambient air by absorbing carbon dioxide from the air and precipitating CaCO_3_.

## 2 Experimental Section

### 2.1 Materials

Methanol, hexane, CaO, and sodium polyphosphate (food additive grade) were purchased from FUJIFILM Wako Pure Chemical Corporation (Osaka, Japan). Congo red and methyl orange were purchased from Nacalai Tesque (Kyoto, Japan). DDT and DEHP were purchased from the Tokyo Chemical Industry (Tokyo, Japan). Trypan blue, PCBs (Aroclor 1254), and scallop shell-derived CaO were purchased from Sigma-Aldrich (Missouri), GL Sciences (Tokyo, Japan), and Plus Lab (Kanagawa, Japan), respectively. *E. coli* (ATCC 51813) was obtained from Microbiologics (Minnesota). Ultrapure water with a resistivity greater than 18.2 MΩ cm at 25 °C was supplied by an RFU464TA Instruments (Advantec, Tokyo, Japan) and used throughout all experiments.

### 2.2 Characterization of CaO Suspensions and Aggregates

CaO was added to ultrapure water at 2 g L^–1^ and vortexed for 1 min (Vortex-Genie 2, Scientific Industries, New York). To the resulting suspensions of the CaO/Ca(OH)_2_ particles, sodium polyphosphate was added at 2 g L^–1^ and vortexed for 1 min. The suspensions underwent flocculation at room temperature.

For optical microscopy, the suspensions were mounted on a glass slide and covered with a coverslip. The samples were observed by using an SZX16 instrument (Olympus, Tokyo, Japan).

For the ζ potential measurement, the suspensions of the CaO/Ca(OH)_2_ particles were resuspended by shaking them by hand just before the measurements. An ELSZ-1000ZS instrument equipped with a flow cell (Otsuka Electronics, Osaka, Japan) was operated at room temperature.

For ultraviolet–visible (UV–vis) spectroscopy, the aggregates of the CaO/Ca(OH)_2_ particles and polyphosphate were resuspended by shaking by hand and left to stand for 30 min before the measurements. The resulting supernatant and suspension of the CaO/Ca(OH)_2_ particles were analyzed using a V-630 instrument (JASCO, Tokyo, Japan) at room temperature.

For the pH measurements, the suspensions were resuspended by shaking by hand just before the measurements. A pH meter F-52 (Horiba, Kyoto, Japan) was operated at room temperature.

For X-ray diffraction (XRD) analysis, the flocculated CaO/Ca(OH)_2_–polyphosphate aggregates were collected by centrifugation, decantation, and freeze-drying. The measurements were carried out using a MiniFlex600 instrument (Rigaku, Tokyo, Japan) equipped with Cu Kα radiation (λ = 1.54 Å). PDXL2 software (version 2.8.4.0, Rigaku, Tokyo, Japan) with International Centre for Diffraction Data (ICDD), Powder Diffraction File (PDF)-4+ 2023, was used to identify peaks of the XRD profiles.

### 2.3 Removal of Dyes

Aqueous solutions of Congo red, trypan blue, and methyl orange were prepared (10, 12, and 25 mg L^–1^, respectively). CaO was added at 2 g L^–1^ to the dye solutions, followed by vortexing for 1 min. After adding sodium polyphosphate at 2 g L^–1^ and vortexing for 1 min, the mixtures were left to stand for 10 min and filtered through qualitative filter paper (Advantec, No. 1). The filtrates were subjected to UV–vis spectroscopy using a V-630 instrument (JASCO, Tokyo, Japan) at room temperature.

### 2.4 Removal of Chemical Pollutants

DDT, DEHP, or PCBs were dissolved in methanol at 10,000 ppm and diluted with ultrapure water to 1, 10, and 100 ppm. To these chemical pollutant solutions (10 mL), CaO was added at 2 g L^–1^ and vortexed for 1 min. Sodium polyphosphate was added at 2 g L^–1^ to the mixed solutions before hand-shaking and vortexing for 1 min. The mixtures were left to stand at room temperature for 10 min and filtered through qualitative filter paper.

For gas chromatography–mass spectrometry (GC–MS), the chemical pollutants contained in 2 mL of the filtrates were extracted using 2 mL of hexane with hand-shaking and vortexing. The extracted solutions were analyzed using a 7890B GC–5977A MS system equipped with an HP-5 ms Ultra Inert GC column (Agilent Technologies, CA), using helium as the carrier gas and an electron ionization ion source. The oven temperature was controlled as follows: 100 °C for 1 min, from 100 to 160 °C at 30 °C min^–1^, from 160 to 270 °C at 5 °C min^–1^, and 270 °C for 3 min. For PCBs, single ion monitoring was performed at 292, 326, and 360 *m*/*z*, which correspond to tetrachlorobiphenyls, pentachlorobiphenyls, and hexachlorobiphenyls, respectively. Electron ionization mass spectra were analyzed using Agilent MassHunter Qualitative Analysis software for chemical species identification. The concentrations of the chemical pollutants were quantified by using calibration curves.

### 2.5 Purification of Model-Contaminated Water

Solutions of DDT, DEHP, and PCBs at 10,000 ppm in methanol were added to 30 mL of water to make the concentration of each chemical species 1 ppm. *E. coli* was added to this solution at 1.6 × 10^6^ colony-forming units (CFUs) mL^–1^, resulting in a model-contaminated water. After scallop shell-derived CaO was added at 2 g L^–1^, the mixtures were mixed using a magnetic stirrer (SW-600H, Nissin, Tokyo, Japan) for 1 min. The mixtures (200 μL) were collected for colony-counting assays using LB agar medium. Meanwhile, sodium polyphosphate was added at 2 g L^–1^ to the mixtures, followed by stirring for 1 min, standing at room temperature for 10 min, and filtration using qualitative filter paper. The filtrate was subjected to GC–MS analyses as described above, pH measurements using an F-52 instrument (Horiba, Kyoto, Japan), and ion chromatography analyses using an IA-300 instrument equipped with a PCI-322 column (DKK-TOA, Tokyo, Japan). Moreover, the pH measurements and ion chromatography analyses were also performed after stirring 20 mL of the filtrate in a 100 mL beaker during exposure to ambient air for 1 and 2 days.

## 3 Results and Discussion

### 3.1 Characterization of CaO Suspensions and Aggregates

We previously reported that the suspensions prepared by adding seashell-derived CaO to water underwent flocculation after the addition of sodium polyphosphate.^34^ In this study, we initially used reagent-grade CaO for fundamental investigations of flocculation with polyphosphate and the removal of chemical pollutants before the demonstration of water purification using seashell-derived CaO.

**Figure 2:**
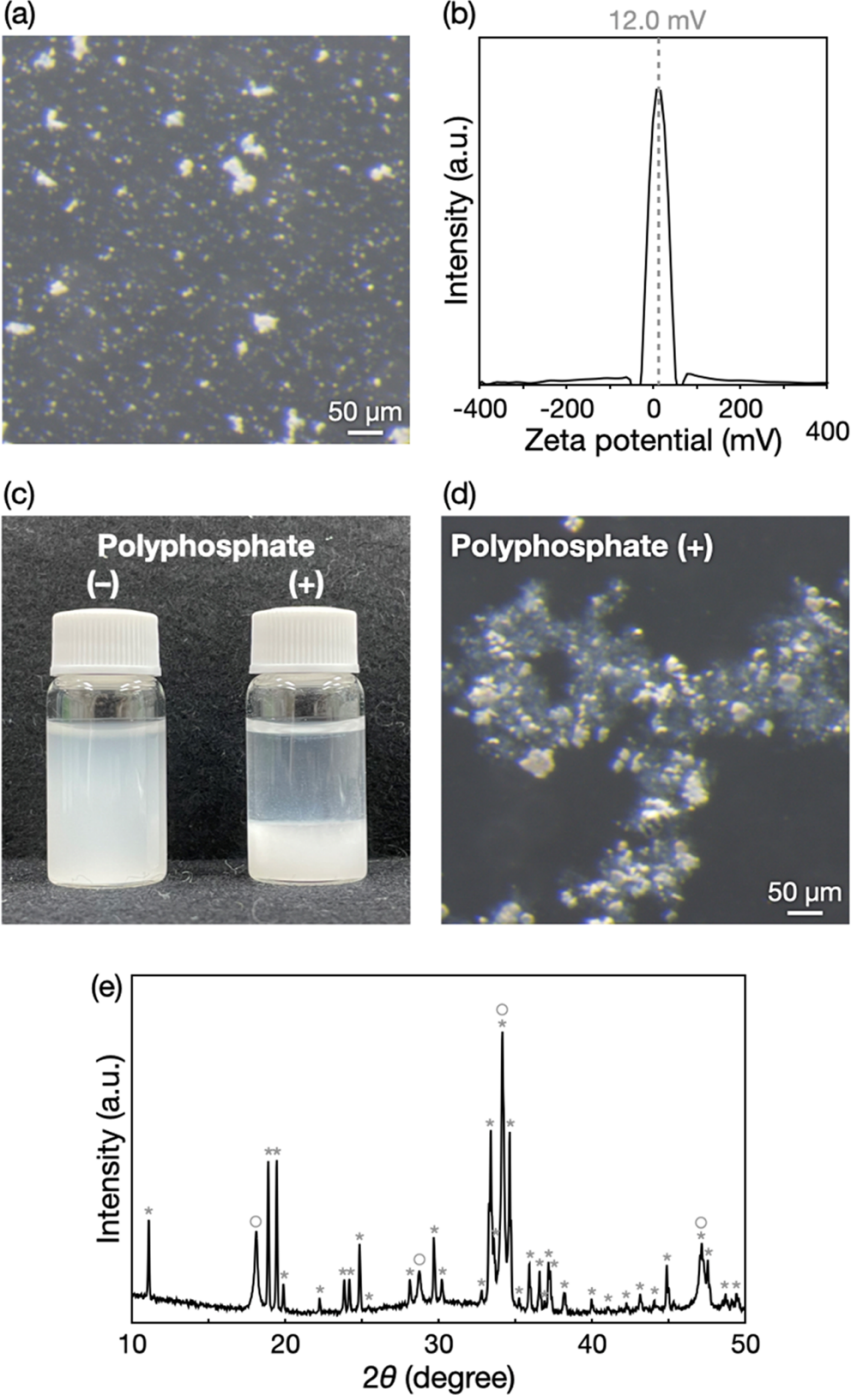
Characterization of CaO/Ca(OH)_2_ suspensions and the aggregates with polyphosphate. (a) Optical microscopy image of CaO/Ca(OH)_2_ suspensions at 2 g L^–1^. (b) ζ Potential of CaO/Ca(OH)_2_ particles. (c) Photograph of CaO/Ca(OH)_2_ suspensions and aggregates with polyphosphate. The samples were shaken by hand for resuspension and left to stand for 1 min before photographing. (d) Optical microscopy image and (e) XRD profile of the aggregates with polyphosphate. The symbols unfilled circle and asterisk in panel (e) denote peaks assignable to crystalline Ca(OH)_2_ and sodium triphosphate phase II, respectively.

CaO is known to rapidly hydrate in water to form Ca(OH)_2_, although this reaction may take a few hours or more to complete.^35,36^ Ca(OH)_2_ has a water solubility of ∼1.6 g L^–1^ at 20 °C.^37^ Thus, in this study, CaO was added to water at 2 g L^–1^ to prepare suspensions of CaO/Ca(OH)_2_ particles (Figure 2a). These particles had a positive charge (Figure 2b), and their zeta potential was measured to be 12.0 ± 0.8 mV (mean ± standard deviation of three individual trials).

The CaO/Ca(OH)_2_ particles underwent flocculation upon the addition of sodium polyphosphate. The pH measurements showed that the salt addition hardly changed the pH of the suspensions from ∼12.5, indicating that the flocculation was not due to changes in pH.^38^ The negatively charged polyphosphate appeared to be adsorbed onto the positively charged CaO/Ca(OH)_2_ particles, causing flocculation via charge neutralization and/or charge-patch interaction.^39,40^ Moreover, the reduction in the double layer around the CaO/Ca(OH)_2_ particles due to the increased ionic strength with the salt addition should also contribute to the flocculation. It appears that the hydrolysis of polyphosphates under alkaline conditions was not significant in this study, given that monomeric phosphate rather increases dispersibility of CaO/Ca(OH)_2_ particles.^41^ The aggregates promptly sedimented within 1 min after resuspension, whereas the CaO/Ca(OH)_2_ particles without polyphosphate took much longer to sediment (Figure 2c). UV–vis spectroscopy revealed that the supernatant was almost transparent (Figure S1), indicating that most of the CaO/Ca(OH)_2_ particles in the suspensions were flocculated by polyphosphate. Microscopic observations indicated fluffy aggregates containing CaO/Ca(OH)_2_ particles and having sizes of more than several tens of micrometers (Figure 2d). The fluffy and voluminous morphology of the aggregates implies the maintenance of a large surface area of CaO/Ca(OH)_2_ particles to a certain extent for adsorbing pollutants. XRD of the aggregates showed peaks assignable to crystalline Ca(OH)_2_ (PDF No. 04–010–3117) and sodium triphosphate phase II (PDF No. 04–009–1422) (Figure 2e); the peak assignments to crystalline Ca(OH)_2_ and sodium triphosphate phase II are shown in Tables S1 and S2, respectively. Other peaks were absent in the XRD profile, indicating the high purity of the materials. Given that the polyphosphate used in this study was polydisperse, the aggregates were considered to contain not only triphosphate but also other phosphate species. In fact, some of the peaks in Figure 2e could also be assigned to sodium phosphate (Table S3).

### 3.2 Removal of Chemical Pollutants by Aggregates

The CaO/Ca(OH)_2_ particles in the aggregates have positive surface charges, which are favorable for adsorbing various anionic species via electrostatic interactions. Moreover, studies have demonstrated that some metal hydroxides act as particulate emulsifiers to stabilize oil–water interfaces, indicating that the surfaces of these metal hydroxides have hydrophobicity to a certain extent.^42−44^ Remarkably, it was reported that the hydrophobicity of a metal hydroxide increased with an increase in pH, especially in the pH region above 12.^42^ Therefore, we hypothesized that the aggregates composed of CaO/Ca(OH)_2_ particles and polyphosphate at pH 12.5 allow the removal of various chemical pollutants via adsorption by electrostatic interactions, van der Waals forces, and hydrophobic effects.

**Figure 3:**
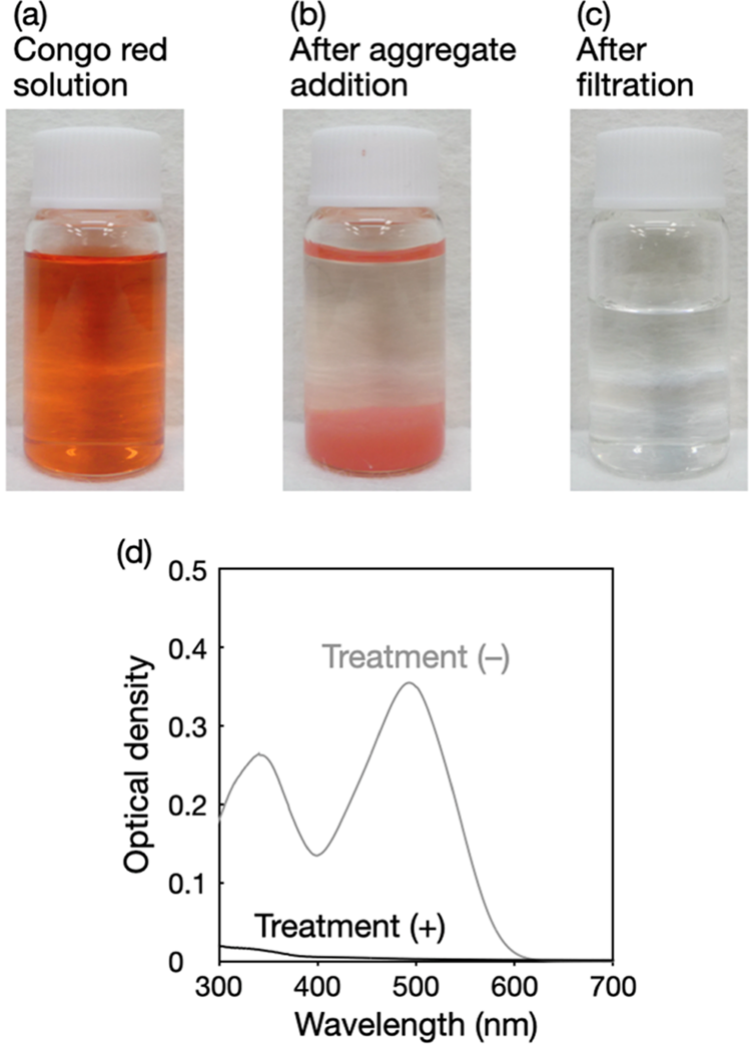
Removal of Congo red by the CaO/Ca(OH)_2_–polyphosphate aggregates. Photographs of the Congo red solutions (a) before and (b) after the addition of the aggregates and (c) subsequent filtration. (d) UV–vis spectra of Congo red solutions before and after the treatment.

An anionic azo dye, Congo red, which is a well-known water pollutant that can cause allergic reactions and be metabolized to carcinogenic product benzidine,^45,46^ was used as a model chemical pollutant. CaO and sodium polyphosphate were added to aqueous Congo red solutions (Figure 3a,b). As a result, red aggregates formed, indicating the adsorption of Congo red by the CaO/Ca(OH)_2_–polyphosphate aggregates formed through the flocculation. The anionic dye was adsorbed by the positively charged CaO/Ca(OH)_2_ particles involving electrostatic interactions. In fact, when Congo red solutions containing CaO/Ca(OH)_2_ particles without polyphosphate were centrifuged, the precipitated CaO/Ca(OH)_2_ particles were red-colored (Figure S2). This result indicates that the CaO/Ca(OH)_2_ particles alone could adsorb Congo red and suggests that the positively charged CaO/Ca(OH)_2_ particles were responsible for the adsorption of the anionic dye by the aggregates. Filtration of the mixtures containing the aggregates with Congo red produced a colorless solution (Figure 3c). The UV–vis spectra of the filtrate exhibited almost no light absorption peaks attributed to Congo red (Figure 3d). These results indicated that CaO/Ca(OH)_2_–polyphosphate aggregates help remove chemical pollutants.

The solutions of other dyes were subjected to treatment using CaO and polyphosphate. Trypan blue, an anionic azo dye, was successfully removed by using CaO/Ca(OH)_2_–polyphosphate aggregates (Figure S3). On the other hand, another anionic azo dye, methyl orange, was hardly removed (Figure S4), suggesting weak interactions between methyl orange and aggregates. Methyl orange with a sulfonate group might have fewer electrostatic interactions with the CaO/Ca(OH)_2_ particles than Congo red and trypan blue, which have two and four sulfonate groups, respectively. Moreover, the lower molecular weight of methyl orange than Congo red and trypan blue might weaken van der Waals forces and hydrophobic effects. Although further investigations are needed to determine the adsorption mechanisms, these results indicate that Congo red and other organic molecules can be removed by CaO/Ca(OH)_2_–polyphosphate aggregates.

We then investigated the removal of representative chemical pollutants with hydrophobic characters.^47^ DDT is one of the world’s best-known and most valuable insecticides.^48^ As DDT and its metabolites are highly toxic and carcinogenic to humans,^49^ the standard value set by the World Health Organization (WHO) for DDT and its metabolites in drinking water is ∼1 ppb.^50^ Methanol solutions of DDT were added to water at 1, 10, and 100 ppm, followed by the addition of CaO and sodium polyphosphate; then, filtration, extraction using hexane, and GC–MS analyses were performed. As the DDT reagent used in this study contained a small amount of impurities, the peak of DDT was subjected to quantification (Figure S5). The DDT concentrations in water before and after treatment are presented in Table 1. These concentrations were significantly decreased by the treatment, indicating the successful removal of the DDT in water. The DDT in water was apparently adsorbed by the CaO/Ca(OH)_2_ particles via van der Waals forces and hydrophobic effects. It was noted that the detection limit in our experiments was ∼10 ppb, which is higher than the standard value for DDT and its metabolites in drinking water (∼1 ppb). Thus, the suitability of the resulting water for drinking was unknown. Nevertheless, the results indicate that CaO/Ca(OH)_2_–polyphosphate aggregates help to remove hydrophobic chemical pollutants such as DDT.

**Table 1:** Removal of Hydrophobic Chemical Pollutants

pollutant	before (ppb)	species	after (ppb)a,b	%removala,b
DDT	1000	dichlorodiphenyltrichloroethane	<10	>99
DDT	10,000	dichlorodiphenyltrichloroethane	<10	>99.9
DDT	100,000	dichlorodiphenyltrichloroethane	102 ± 22	99.90 ± 0.02
DEHP	1000	di(2-ethylhexyl)phthalate	<10	>99
DEHP	10,000	di(2-ethylhexyl)phthalate	<10	>99.9
DEHP	100,000	di(2-ethylhexyl)phthalate	38.2 ± 10.0	99.96 ± 0.01
PCBs	1000	2,2′,3,5′-tetrachloro-1,1′-biphenyl		>90
		2,3,3′,4,4′-pentachloro-1,1′-biphenyl		>90
		2,2′,4,4′,5,5′-hexachloro-1,1′-biphenyl		>90
PCBs	10,000	2,2′,3,5′-tetrachloro-1,1′-biphenyl		98.1 ± 0.5
		2,3,3′,4,4′-pentachloro-1,1′-biphenyl		>99
		2,2′,4,4′,5,5′-hexachloro-1,1′-biphenyl		>99
PCBs	100,000	2,2′,3,5′-tetrachloro-1,1′-biphenyl		99.68 ± 0.04
		2,3,3′,4,4′-pentachloro-1,1′-biphenyl		>99.9
		2,2′,4,4′,5,5′-hexachloro-1,1′-biphenyl		>99.9

a,b The values are expressed as mean ± standard deviation for three individual trials.

The removal of other kinds of hydrophobic chemical pollutants was investigated. DEHP is primarily used as a plasticizer and is possibly carcinogenic to humans.^50^ The DEHP reagent used in this study exhibited a single peak in the chromatogram of GC–MS (Figure S6). Treatment with CaO and polyphosphate decreased the DEHP concentrations in water from 1 and 10 ppm to <10 ppb (Table 1). This value is comparable to that set by the WHO for DEHP in drinking water, which is ∼8 ppb.^50^ PCBs are a group of chlorinated aromatic hydrocarbons differing in the number and positions of chlorine atoms and are known as water pollutants.^47^ Although PCBs were used primarily as electrical insulating, heat transfer, and lubricating fluids,^51^ their production was banned due to their toxicity.^52^ The chromatogram of the PCB reagent by GC–MS is presented in Figure S7. The use of CaO/Ca(OH)_2_–polyphosphate aggregates led to a significant decrease in PCB concentrations (Table 1). It was noted that the absolute concentration of each species of PCBs could not be quantified. Collectively, it was found that the flocculation of CaO/Ca(OH)_2_ particles with polyphosphate leads to the removal of various chemical pollutants, including negatively charged dyes and hydrophobic molecules, in water.

### 3.3 Water Purification via Disinfection and Flocculation

We performed water purification using CaO and polyphosphate, including a disinfection step and a subsequent flocculation step for chemical pollutant removal (Figure 1). It is noted that CaO exhibits good bactericidal activity at concentrations higher than its saturation concentration.^28^ Therefore, in a practical situation, the saturation of CaO/Ca(OH)_2_ would serve as a visual indicator of disinfection. In this study, a model-contaminated water was prepared by adding *E. coli* and chemical pollutants, namely, DDT, DEHP, and PCBs, to ultrapure water at 1.6 × 10^6^ CFU mL^–1^ and 1, 1, and 1 ppm, respectively. Moreover, seashell-derived CaO rather than reagent-grade CaO was used to bring this experiment closer to practical application.

**Figure 4:**
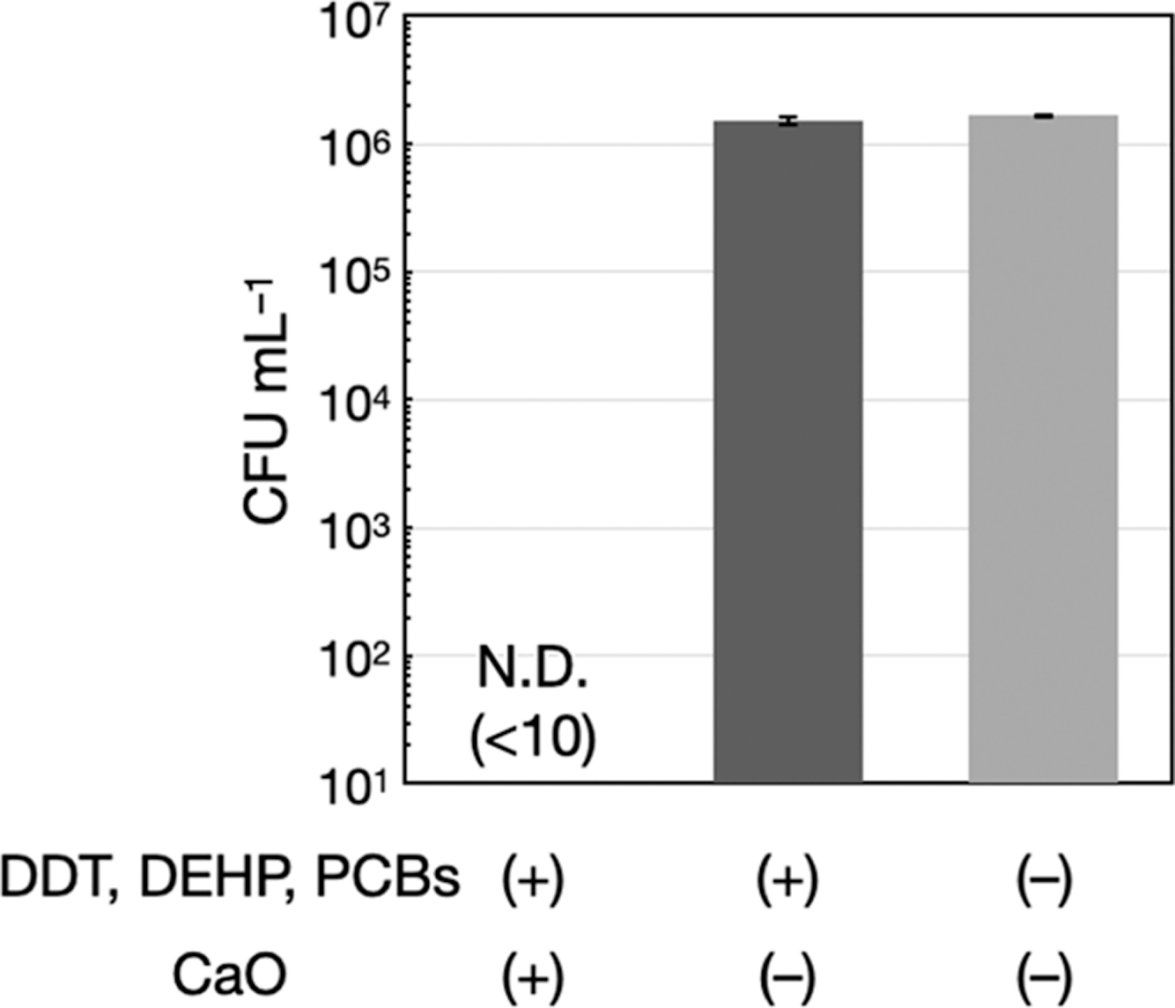
Disinfection of the model-contaminated water with seashell-derived CaO. CFU mL^–1^ values of *E. coli* before and after the addition of chemical pollutants (DDT, DEHP, and PCBs) and seashell-derived CaO are presented. N.D. denotes not detected (CFU mL^–1^ < 10). The error bars represent the standard deviation of three individual trials.

The amounts of viable *E. coli* in the model-contaminated water before and after the treatment with seashell-derived CaO were evaluated by the colony-counting method. The addition of seashell-derived CaO at 2 g L^–1^ to the model-contaminated water resulted in a >99.999% reduction in *E. coli* counts (Figure 4). It was noted that the chemical pollutants caused no change in the CFU of *E. coli*, indicating that seashell-derived CaO was responsible for the bactericidal action. This high bactericidal activity of seashell-derived CaO is consistent with the results from previous reports.^10,11^

Sodium polyphosphate was added to the model-contaminated water after disinfection by seashell-derived CaO for flocculation, followed by filtration. As a result, the concentrations of DDT, DEHP, and PCBs successfully decreased (Table 2). It was noted that the extent of decrease in DDT concentration was smaller than when using reagent-grade CaO for water containing DDT alone (i.e., without DEHP, PCBs, and *E. coli*) (Table 1). The possible explanations for this difference are that seashell-derived CaO had fewer adsorption sites for DDT than reagent-grade CaO and that the adsorption of DDT competed with that of DEHP, PCBs, and biomolecules from dead *E. coli*. Although removal efficiency might vary depending on the CaO origin or the total amount of contaminants, the method of chemical pollutant removal using CaO and polyphosphate was shown to be operative even for water contaminated with various chemical pollutants and bacteria.

**Table 2:** Removal of Chemical Pollutants from the Model-Contaminated Water

pollutant	before (ppb)	species	after (ppb)a,b	%removala,b
DDT	1000	dichlorodiphenyltrichloroethane	31 ± 3	96.9 ± 0.3
DEHP	1000	di(2-ethylhexyl)phthalate	<10	>99
PCBs	1000	2,2′,3,5′-tetrachloro-1,1′-biphenyl		>90
		2,3,3′,4,4′-pentachloro-1,1′-biphenyl		>90
		2,2′,4,4′,5,5′-hexachloro-1,1′-biphenyl		>90

a,b The values are expressed as mean ± standard deviation for three individual trials.

**Figure 5:**
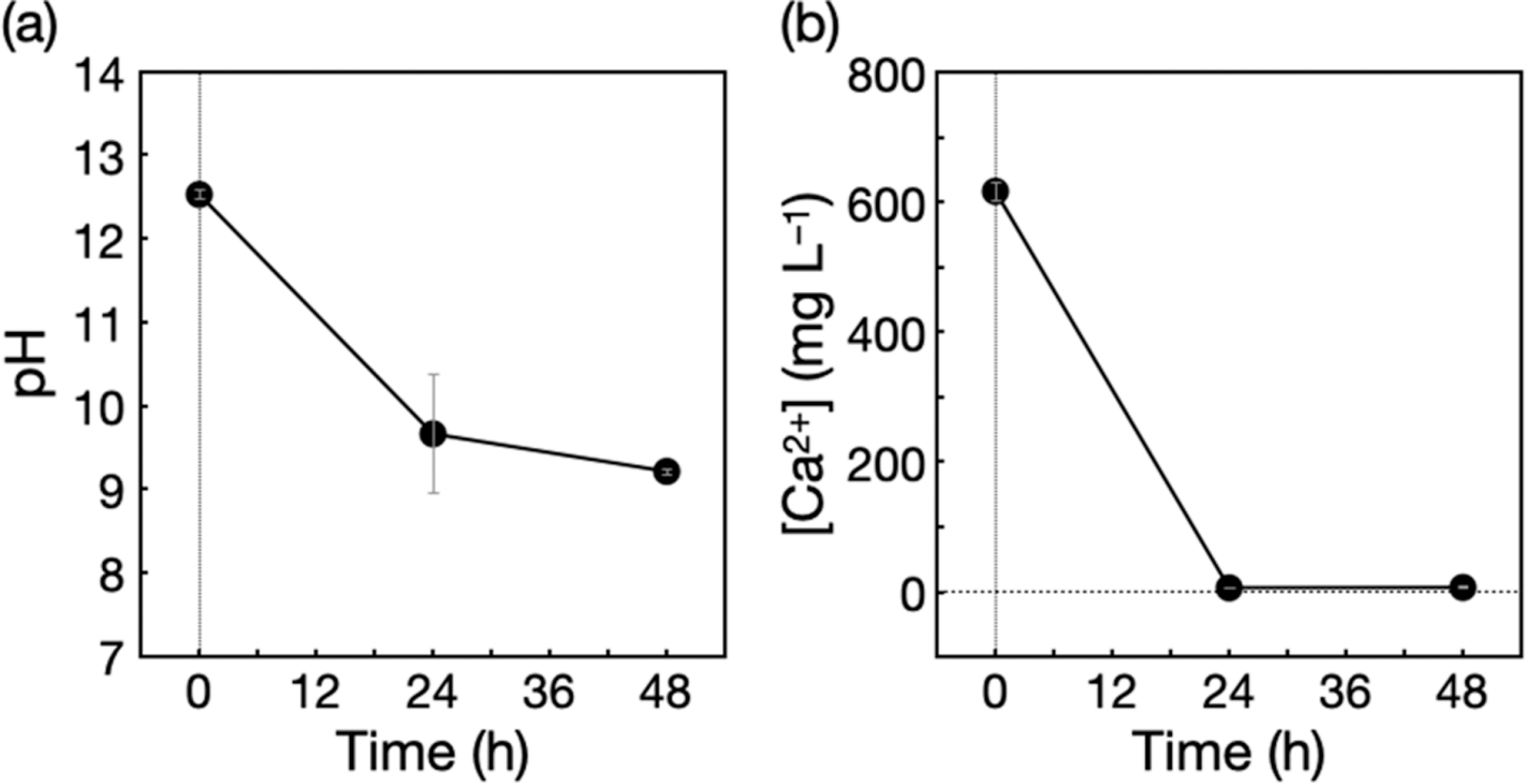
Decreases in (a) pH and (b) Ca^2+^ concentration of the filtrate during exposure to ambient air with stirring.

The water purified through disinfection by CaO, flocculation with polyphosphate, and filtration has high pH and Ca^2+^ concentrations that make it inappropriate for drinking, medical uses, and other purposes. In fact, long-term exposure to alkaline drinking water has been suggested to have profound systemic effects, such as significant growth retardation.^53^ In this context, the pH and Ca^2+^ concentrations in water purified using CaO were found to decrease only by exposing the water to ambient air. Figure 5a presents the time course of the pH of the filtrate during exposure to ambient air with stirring, showing a pH decrease in the weak alkaline region. In addition, the production of white precipitates was observed in the filtrate. These results indicate that the filtrate absorbed carbon dioxide from air, which contained carbon dioxide at 0.04%, to decrease pH by producing insoluble CaCO_3_.^29^ In fact, ion chromatography analyses revealed that the Ca^2+^ concentration in the filtrate decreased (Figure 5b), which supports this conjecture. The resulting Ca^2+^ concentration was 6–7 mg L^–1^, which was within the Ca^2+^ concentrations in tap water.^54^ This characteristic of CaO, i.e., easy removal after use, is different from the characteristics of common disinfectants and even sodium hydroxide; common disinfectants tend to remain after disinfection, and sodium ion from sodium hydroxide hardly precipitates with carbonate ions and other anions. Overall, seashell-derived CaO has great potential for the production of safe water.

## 4 Conclusions

In this study, we proposed water purification using seashell-derived CaO through disinfection, flocculation with polyphosphate, filtration, and subsequent exposure to ambient air. Seashell-derived CaO, which has been investigated as a disinfectant, successfully acted as an adsorbent when forming aggregates with polyphosphate, removing the chemical pollutants from the water. The results in this study indicate the advantages of the water purification strategy using CaO and polyphosphate: first, the saturation of CaO/Ca(OH)_2_ potentially serves as a visual indicator of disinfection without using any instruments for viable bacterial detection. Second, the flocculation by polyphosphate removes excess CaO/Ca(OH)_2_ as well as chemical pollutants. Third, the high pH and Ca^2+^ concentrations in the resulting purified water are readily decreased by exposure to ambient air, leading to the maintenance of the pH and Ca^2+^ concentration suitable for drinking and other purposes. It should be highlighted that both of the materials used, the seashell-derived CaO and sodium polyphosphate, are widely used as food additives. While this water purification method produces a certain amount of polluted waste, it requires no special equipment or techniques and thus is expected to be especially useful under emergency conditions due to disasters, where safe water supply is crucial,^55−57^ by allowing the purification of surface water containing chemical pollutants and harmful microorganisms. Further investigations using surface water from rivers, lakes, or ponds will reveal the practical potential of this water purification method. Collectively, our findings promote the development of seashell-derived material–polymer complexes for water purification.

## Supporting Information Available

UV–vis spectra of CaO/Ca(OH)_2_ suspensions and supernatant after polyphosphate addition; XRD of Ca(OH)_2_, sodium triphosphate phase II, and sodium phosphate; photographs and UV–vis spectra of dye solutions; and representative chromatograms and mass spectra of chemical pollutants (PDF)
