# Supplementary material for: Sequential Approach for Water Purification Using Seashell-Derived Calcium Oxide through Disinfection and Flocculation with Polyphosphate for Chemical Pollutant Removal
Source: ACS Omega. 2024 Mar 6;9(11):12635–42. doi: 10.1021/acsomega.3c07627 (PMC10955710; doi:10.1021/acsomega.3c07627)
Supplement: Supplementary file 1 — ao3c07627_si_001.pdf [file ao3c07627_si_001.pdf]

Supporting Information

Sequential Approach for Water Purification using Seashell-Derived Calcium Oxide through Disinfection and Flocculation with Polyphosphate for Chemical Pollutant Removal

Yuuki Hata,[†,‡] Sumiyo Hiruma,[‡] Hiromi Miyazaki,[‡] and Shingo Nakamura*[‡]*

[†]Department of Chemical Science and Engineering, School of Materials and Chemical Technology, Tokyo Institute of Technology, 2-12-1-H121 Ookayama, Meguro-ku, Tokyo 152-8550, Japan

[‡]Division of Biomedical Engineering, National Defense Medical College Research Institute, 3-2 Namiki, Tokorozawa-shi, Saitama 359-8513, Japan

* Correspondence: hata@mac.titech.ac.jp (Y.H.); snaka@ndmc.ac.jp (S.N.)

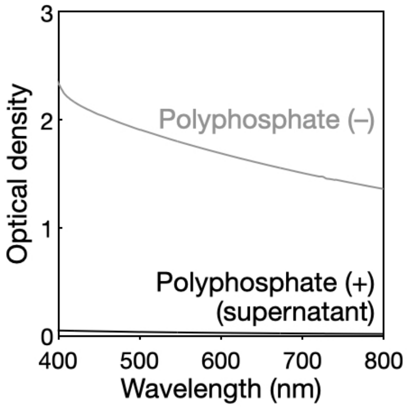

Figure S1. Ultraviolet–visible (UV–Vis) spectra of calcium oxide (CaO)/calcium hydroxide ($Ca(OH)_2$) suspensions and supernatant after flocculation by sodium polyphosphate.

Table S1. X-Ray Diffraction (XRD) of $Ca(OH)_2$ (Powder Diffraction File (PDF) No. 04-010-3117)

2θ (degree)[a]	h k l
18.05	0 0 1
28.70	1 0 0
34.11	1 0 1
47.13	1 0 2

[a]The values are calculated based on Cu Kα radiation (λ = 1.54059 Å).

Table S2. XRD of Sodium Triphosphate Phase II (PDF No. 04-009-1422)

2θ (degree)[a]	h k l	2θ (degree)[a]	h k l	2θ (degree)[a]	h k l
11.07	2 0 0	33.42	-5 1 1	40.87	3 1 4
18.81	-2 0 2	33.63	6 0 0	41.75	-4 2 1
19.37	-1 1 1	34.14	5 1 1	42.22	4 2 1
19.77	2 0 2	34.37	2 0 4	43.20	7 1 0
22.24	4 0 0	34.50	3 1 3	43.90	-1 1 5
23.80	3 1 0	35.14	0 2 1	44.38	7 1 1
24.06	1 1 2	35.92	-5 1 2	44.80	4 2 2
24.82	-3 1 1	36.44	-1 1 4	45.37	8 0 0
25.38	3 1 1	36.82	-2 2 1	47.02	7 1 2
28.06	4 0 2	37.09	2 2 1	47.41	0 2 4
29.59	-1 1 3	37.25	5 1 2	48.42	-2 2 4
30.07	1 1 3	38.16	6 0 2	48.72	6 2 0
32.80	5 1 0	39.39	-2 2 2	49.17	-6 2 1
33.22	-3 1 3	39.97	-5 1 3	49.27	2 2 4

[a]The values are calculated based on Cu Kα radiation (λ = 1.54059 Å).

Table S3. XRD of Sodium Phosphate (PDF No. 00-011-0652)

2θ (degree)[a]	h k l	2θ (degree)[a]	h k l	2θ (degree)[a]	h k l
11.02	0 0 2	32.75	0 1 5	37.93	2 2 0
18.79	-2 0 2	33.23	-3 1 3	38.15	2 0 6
19.41	-1 1 1	33.33	-4 0 2	39.42	-4 1 3
19.85	2 0 2	33.55	0 0 6	39.93	-3 1 5
22.21	0 0 4	34.10	1 1 5	40.28	4 0 4
23.77	0 1 3	34.22	0 2 0	41.03	4 1 3
24.16	2 1 1	34.58	3 1 3	41.72	-1 2 4
24.85	-1 1 3	35.18	1 2 0	42.26	1 2 4
25.43	1 1 3	35.85	-2 1 5	43.12	0 1 7
26.59	-2 0 4	36.56	-4 1 1	43.47	-1 1 7
28.08	2 0 4	36.82	-1 2 2	44.05	3 2 2
29.66	-3 1 1	37.10	4 1 1	44.86	2 2 4
30.18	3 1 1	37.28	2 1 5		

[a]The values are calculated based on Cu Kα radiation (λ = 1.54059 Å).

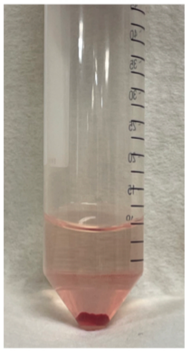

Figure S2. Congo red solutions containing $CaO/Ca(OH)_2$ particles after centrifugation.

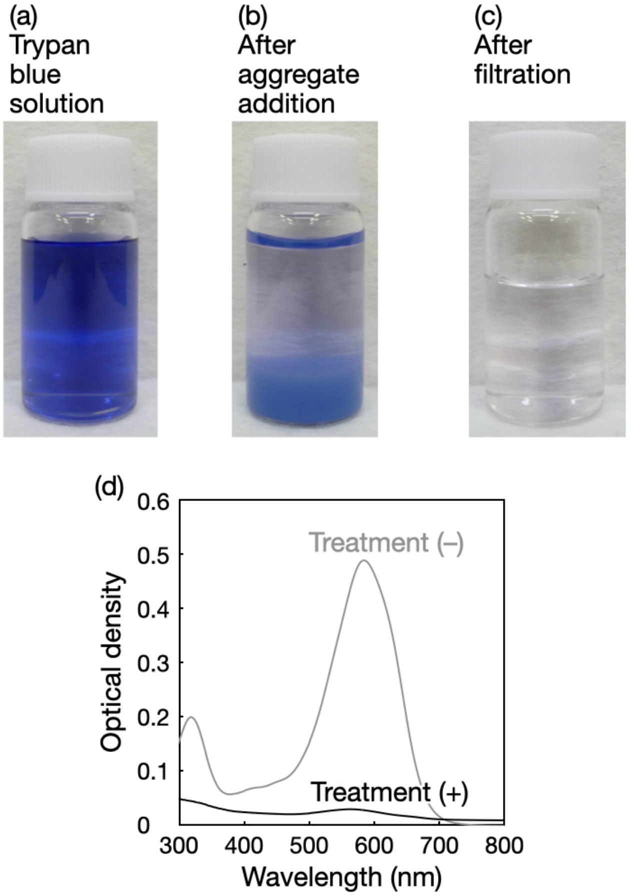

Figure S3. The treatment using CaO and polyphosphate for trypan blue solutions. Photographs of trypan blue solutions (a) before and (b) after the addition of $CaO/Ca(OH)_2$–polyphosphate aggregates and (c) subsequent filtration. (d) UV–Vis spectra of trypan blue solutions before and after the treatment.

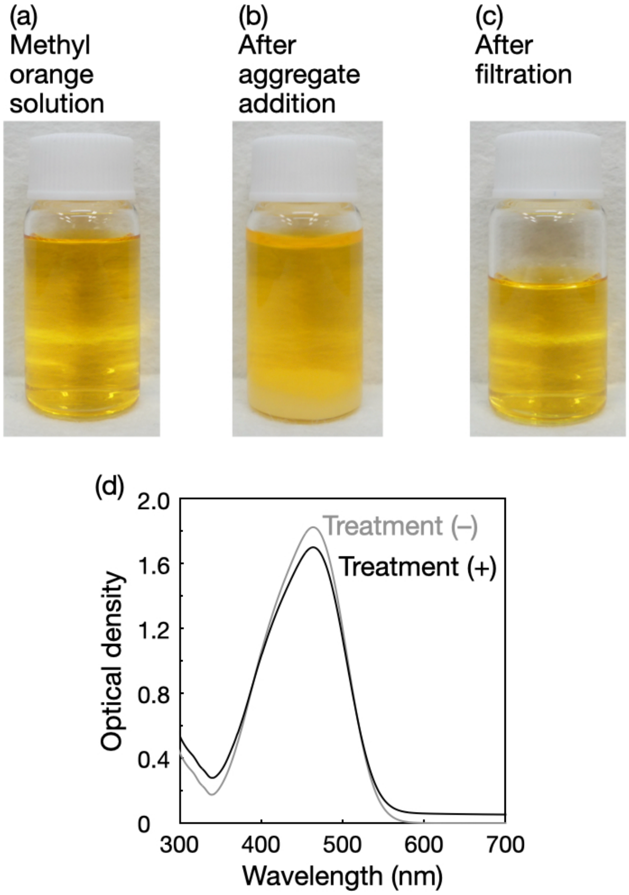

Figure S4. The treatment using CaO and polyphosphate for methyl orange solutions. Photographs of methyl orange solutions (a) before and (b) after the addition of $CaO/Ca(OH)_2$–polyphosphate aggregates and (c) subsequent filtration. (d) UV–Vis spectra of methyl orange solutions before and after the treatment.

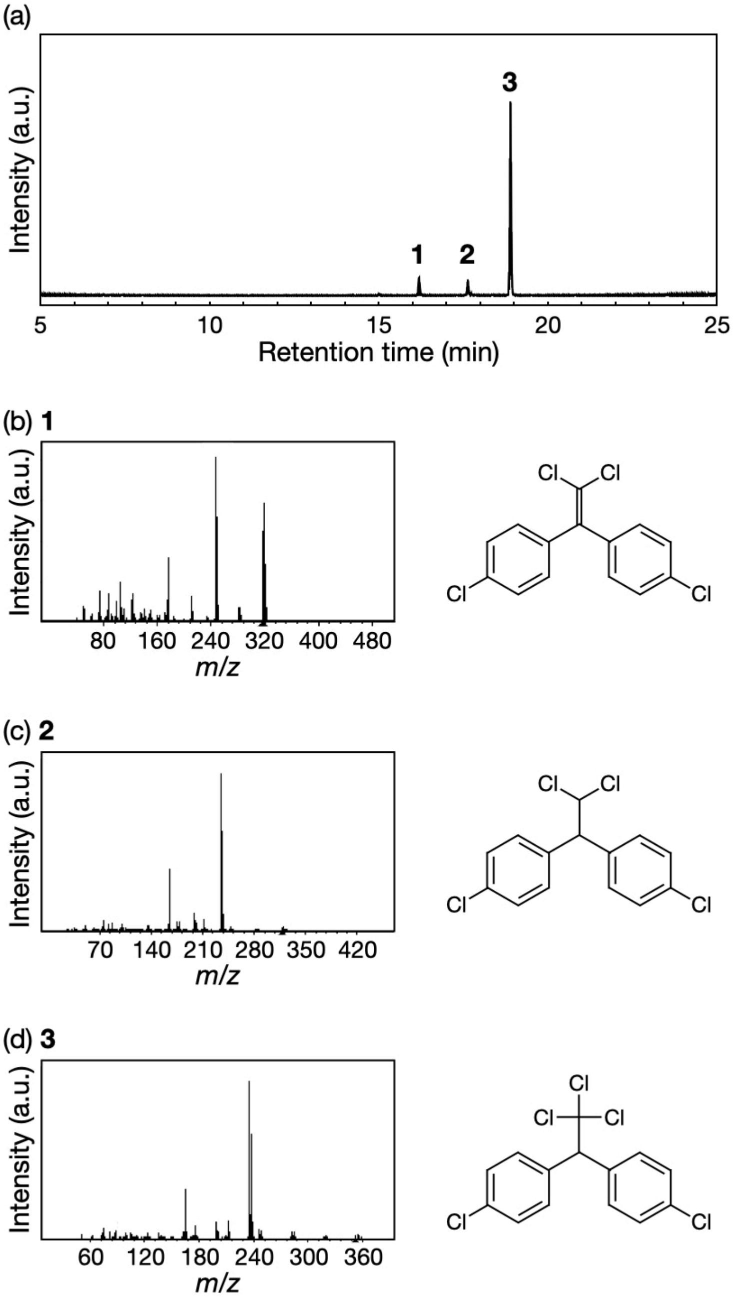

Figure S5. Gas chromatography–mass spectrometry (GC–MS) analyses of dichlorodiphenyltrichloroethane. (a) Representative chromatogram. (b–d) Electron ionization mass spectra of the three peaks observed in (a). The chemical structures of the identified species are shown.

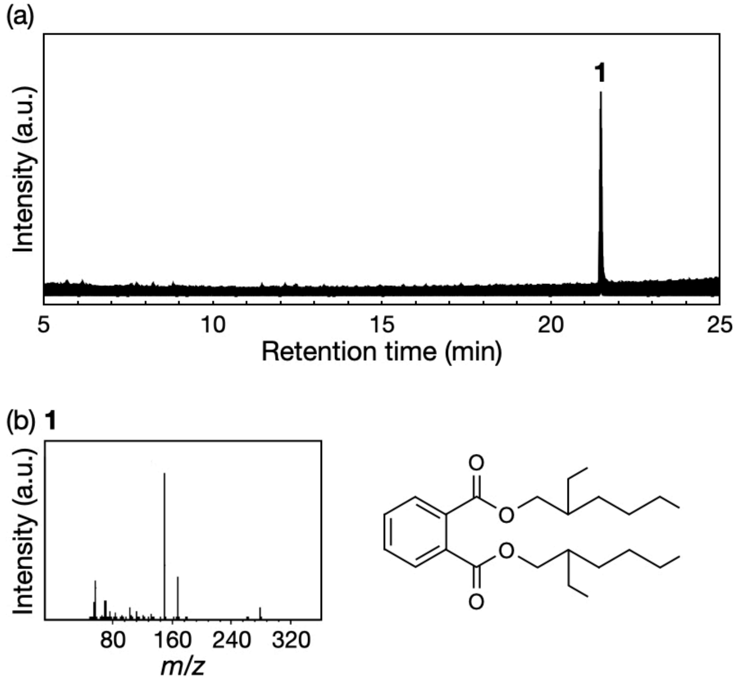

Figure S6. GC–MS analyses of di(2-ethylhexyl)phthalate. (a) Representative chromatogram. (b) Electron ionization mass spectrum of the peak observed in (a). The chemical structure of the identified species is shown.

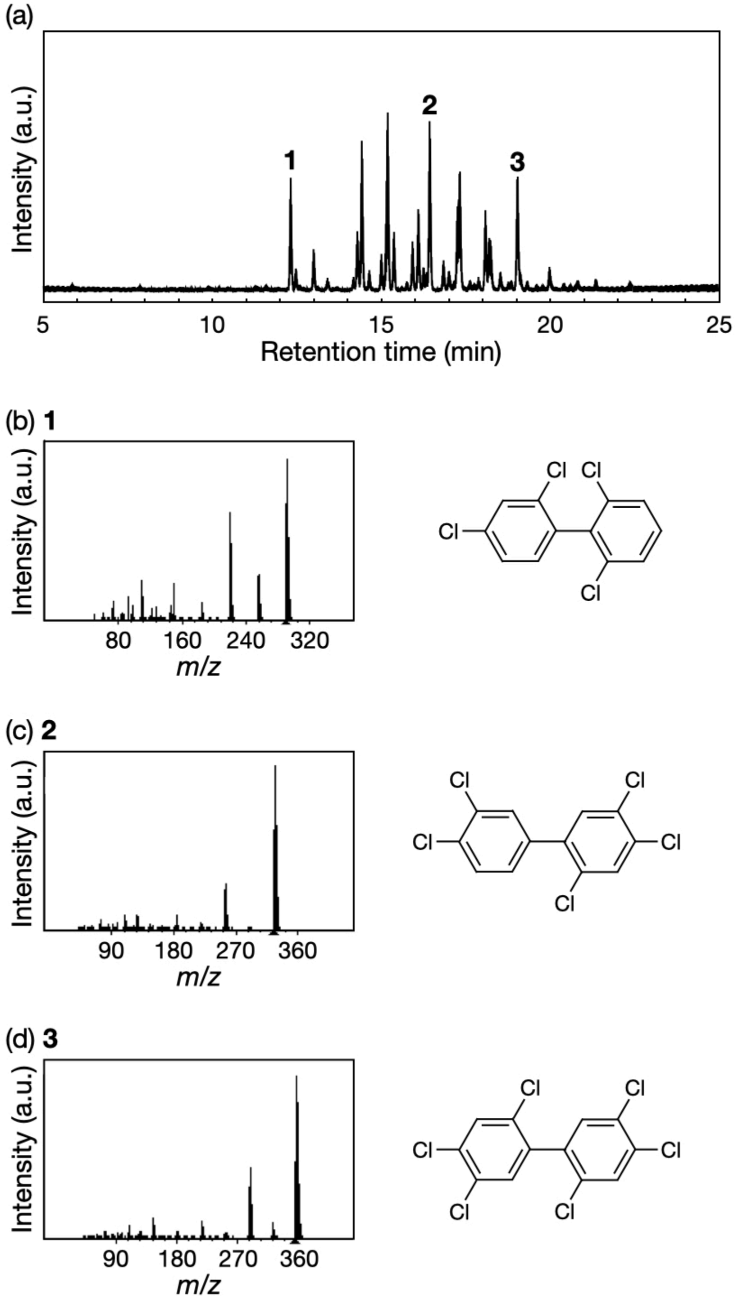

Figure S7. GC–MS analyses of polychlorinated biphenyls. (a) Representative chromatogram. (b–d) Electron ionization mass spectra of three peaks observed in (a). The chemical structures of the identified species are shown.
